# Genetic polymorphisms of IL-17A, IL-17F, TLR4 and miR-146a in association with the risk of pulmonary tuberculosis

**DOI:** 10.1038/srep28586

**Published:** 2016-06-24

**Authors:** Min Wang, Guisheng Xu, Lingshuang Lü, Kun Xu, Yongzhong Chen, Hongqiu Pan, Bo Burstrom, Kristina Burstrom, Jianming Wang

**Affiliations:** 1 Department of Epidemiology, School of Public Health, Nanjing Medical University, Nanjing, 211166, China; 2 Department of Social Medicine and Health Education, School of Public Health, Nanjing Medical University, Nanjing, 211166, China; 3 Department of Tuberculosis, Third Hospital of Zhenjiang City, Zhenjiang, 212005 PR China; 4 Department of Public Health Sciences, Karolinska Institutet, 171 77 Stockholm, Sweden; 5 Health Care Services, Stockholm County Council, 171 77 Stockholm, Sweden; 6 Department of Learning, Informatics, Management and Ethics, Karolinska Institutet, 171 77 Stockholm, Sweden; 7 The Innovation Center for Social Risk Governance in Health, School of Public Health, Nanjing Medical University, Nanjing, 211166, China

## Abstract

Genetic factors affect host susceptibility to pathogens. In this population-based case control study, we explored the genetic polymorphisms of IL-17, TLR4 and miR-146a in association with pulmonary tuberculosis in a Chinese Han population. We recruited 1601 pulmonary tuberculosis patients matched with 1526 healthy controls and genotyped twelve functional single nucleotide polymorphisms (SNPs). After the correction for multiple comparisons, two SNPs (rs10759932 and rs2737190) in the TLR4 gene remained significant. Individuals carrying the rs2737190-AG genotype (vs. AA) had a significantly increased risk of either clinical tuberculosis (OR: 1.31, 95% CI: 1.11–1.53) or sputum smear-positive tuberculosis (OR: 1.35, 95% CI: 1.13–1.61). Stratification analysis revealed that the effects of genetic variations on tuberculosis were more evident among non-smokers. People with haplotype TLR4 rs10983755G–rs10759932C had a significantly increased risk of tuberculosis (OR: 3.43, 95% CI: 2.34–5.05). Moreover, we found that SNPs of rs3819024 in IL-17A and rs763780 in IL-17F were weakly related to a prognosis of tuberculosis. Our results suggest that genetic polymorphisms of IL-17 and TLR4 may play a role in host susceptibility to tuberculosis in the Chinese Han population. More work is necessary to identify specific causative variants of tuberculosis underlying the observed associations.

Tuberculosis is a chronic infectious disease caused by the pathogen of *Mycobacterium tuberculosis* (MTB), and has been a major public health problem worldwide^1^. An estimated 9 million people developed active tuberculosis, and 1.5 million died from it in 2013, mostly in developing countries^2^. The outcome of MTB infection ranges from complete pathogen clearance to asymptomatic latent infection to active tuberculosis disease. Most infected individuals are in the latent period, and only 5–10% will progress to the active phase during their lifetimes^3^,^4^,^5^. Researchers have shown that the innate and adaptive immune responses play an important role in the control of MTB infection^6^.

CD4(+) T cells play a critical role during MTB infection by regulating the immune response and mediating host protection. Th1 and Th17 cells are the main effector CD4(+) T cells^7^. Th1 cells contribute to tuberculosis protection by secreting IFN-γ and activating the antimycobacterial reaction in macrophages^7^. Th17 cells are interleukin (IL)-17-producing CD4^+^ T cells with implications in inducing neutrophilic inflammation and mediate tissue damage^7^,^8^. Antimicrobial inflammatory response primarily begins through the initial sensing of different pathogen-associated molecular patterns by the pattern recognition receptors of the host^9^. Amongst the innate immune receptors, Toll-like receptors (TLRs) have the unique capacity to sense the initial infection and are the most potent inducers of the immune responses^9^. Toll-like receptor 4 (TLR4) is the main receptor mediating the signals responsible for the production of IL-17A induced by MTB^10^. The deficiency of TLR4 inhibits Th17 cell differentiation by suppressing the Signal Transducer and Activator of Transcription 3 (STAT3) pathway and promoting Th1 cell differentiation by enhancing the STAT1 pathway^11^. As shown in Fig. 1, microRNA-146a (miR-146a) is also involved in the host immune response to MTB infection by acting as a negative feedback regulator of the TLR/NF-kB pathway and potentially participating in regulating IL-17 expression by targeting the 3′-untranslated region (UTR) of the TRAF6 and the IRAK-1 genes^12^,^13^. The activation of innate immunity receptors via a pathogen induces the up-regulation of miR-146a expression and will in turn exert a negative feedback on TLR4, leading to an inhibition of Th17 pathway molecules and pro-inflammatory cytokines (IL-17A, IL-17F, IL-6 and TNF-α) and an attenuation of the inflammatory effect of Th17 cells^12^.

Both IL-17A and IL-17F are members of the IL-17 cytokine family. They are located adjacent to one another on the same human chromosome, 6p12, and have similar expression profiles^14^. The TLR4 gene is located on the long arm of chromosome 9 at position 33.1^15^. Although genetic polymorphisms of IL-17 and TLR4 have gained much more interest in the risk of tuberculosis^16^,^17^,^18^,^19^,^20^, few studies have examined their synergistic effect, and a small number of these studies were performed in China. Considering the roles of TLR4, IL-17 and miR-146a in the pro-inflammatory response^12^, we conducted a population-based case control study in a Chinese Han population, with the goals of exploring whether genetic polymorphisms in IL-17, TLR4, and miR-146a are associated with susceptibility to and the prognosis of pulmonary tuberculosis.

## Materials and Methods

### Study design and study population

This study has a mixed case control and prospective follow-up design. We recruited 1601 pulmonary tuberculosis patients from Jiangsu province, China since 2011. They were genetically-unrelated Chinese Han individuals. Patients were aged 18 years or older, without HIV infection, cancer or autoimmune diseases. Tuberculosis cases were group-matched (by sex and age) with 1526 controls from a pool of individuals who participated in the community-based health examination programs. Individuals with a history of tuberculosis, diabetes, malignancy, HIV and immunosuppressive conditions were excluded. This study was approved by the ethics committee of Nanjing Medical University (No: 2012-0105, Date: Jan 5, 2012). The methods were carried out in accordance with the approved guidelines. Written informed consent was obtained from all participants. The manuscript was drafted according to the STROBE statement (http://www.strobe-statement.org/).

### Diagnosis of tuberculosis

Tuberculosis cases were diagnosed by specialized doctors following the guidelines recommended by the China Ministry of Health, which were based on clinical symptoms and signs, chest x-ray examination, sputum smear tests or sputum culture (http://www.chinatb.org). Three sputum samples were collected from each subject with labelled plastic bottles. The Ziehl-Neelsen hot staining method was used for sputum smear microscopy. If the equipment and technology allowed, the culture was carried out. In brief, sputum samples were decontaminated with 4% sodium hydroxide (NaOH), centrifuged and then cultured on Lowenstein-Jensen (LJ) culture media^21^. The LJ culture media were incubated at 37 °C. Identification of MTB was done using the p-nitrobenzoic acid (PNB) and thiophene carboxylic acid hydrazine (TCH) resistance test. Growth in LJ medium containing PNB indicates that the bacilli do not belong to the MTB complex. Species other than MTB were excluded from the current analysis.

### Data collection

Trained local health facility staff interviewers administered a risk factor questionnaire to all participants. The collected data included demographic characteristics, tobacco smoking, alcohol drinking, medical history and laboratory tests. Patients were followed to obtain information on their therapeutic regimens, treatment adherence and outcomes. After informed consent was obtained, a blood sample was collected from each participant for molecular analyses.

### SNP selection and genotyping

We selected SNPs in the IL-17 and TLR4 genes based on the following criteria: (1) minor allele frequency (MAF) ≥ 0.05 in the Chinese Han population; (2) Hardy-Weinberg equilibrium test: P ≥ 0.05; and (3) SNPs located in the functional areas such as 5′-UTR, 5′ near the gene, exon or 3′-UTR. In addition, a functional polymorphism in the miR-146a gene was also selected for genotyping (http://www.bioguo.org/miRNASNP2/). As a result, twelve SNPs were genotyped, including four SNPs in IL-17A (rs2275913, rs3819024, rs8193036 and rs3748067), one SNP in IL-17F (rs763780), six SNPs in TLR4 (rs1927914, rs10759932, rs2737190, rs10983755, rs7873784, rs11536889) and one SNP in miR-146a (rs2910164). Genomic DNA was extracted from leukocytes in the peripheral blood sample by proteinase K digestion and phenol/chloroform extraction. The primer and probe sequences for each SNP were showed in Table 1. According to the manufacturer’s instructions, we genotyped SNPs using the TaqMan allelic discrimination technology on the 384-well ABI 7900HT Real-Time PCR System (Applied Biosystems, Foster City, CA, USA), ascertained using SDS software (version 2.3)^22^. Amplification was performed under the following conditions: 50 °C for 2 min, 95 °C for 10 min followed by 45 cycles of 95 °C for 15 s and 60 °C for 1 min. The success rate for each SNP was over 96%. To avoid batch bias, we allocated DNA samples of both cases and controls in each plate with no discrepancies between the reaction conditions. Approximately 10% of the samples were randomly selected for repeat genotyping for confirmation, and the results were 100% concordant.

### Statistical analysis

Data were entered with EpiData 3.1 software (Denmark) and analyzed using STATA 10.0 (StataCorp, College Station, TX, USA). Student’s t-test (for continuous variables) and the χ^2^ test (for categorical variables) were used to analyze the differences in demographic variables and potential risk factors between cases and controls. Hardy-Weinberg equilibrium (HWE) was tested using a goodness-of-fit χ^2^ test by comparing the observed genotype frequencies with the expected frequencies among the controls to make sure that the alleles were independently segregated. An unconditional logistic regression model was carried out to analyze the associations between genotypes and the risk of tuberculosis by calculating the odds ratio (OR) and 95% confidence interval (CI). The relative risk (RR) and 95% CI were calculated to evaluate the effect of genetic polymorphisms on the patient prognoses. To control for potential confounding, we adjusted for age, sex, tobacco smoking and alcohol drinking. To comprehensively analyze the effect of SNPs, we applied three different genetic models: additive model, dominant model and recessive model. IL-17A and TLR4 haplotypes were performed using phase 2.1 software. Bonferroni corrections were applied for multiple comparisons.

## Results

### General characteristics of the study subjects

Demographic characteristics of the cases and controls are shown in Table 2. In total, 1601 tuberculosis cases (73.8% males and 26.2% females) and 1526 controls (72.9% males and 27.1% females) were recruited. The average (±standard deviation, SD) age was 52.1(±17.7) years in cases and 52.4(±17.0) years in controls. Due to the frequency matching, there was no significant difference in the distribution of age and sex between the two groups. The proportion of ever smokers was 52.4% among cases, which was significantly higher than that in controls (36.0%) (*χ*^*2*^* = *84.73, P < 0.001). Alcohol drinking was inversely related to tuberculosis, and 22.2% of the cases vs. 26.8% of the controls had a history of alcohol consumption (*χ*^*2*^* = *9.06, P = 0.003).

### Risk analysis

Except for rs1927914 (P = 0.012), the genotype distributions of the eleven SNPs were all in HWE in the controls (P = 0.43 for rs2275913, P = 0.41 for rs3819024, P = 0.84 for rs8193036, P = 0.12 for rs3748067, P = 0.06 for rs763780, P = 0.10 for rs10759932, P = 0.07 for rs2737190, P = 0.34 for rs10983755, P = 0.60 for rs7873784, P = 0.98 for rs11536889 and P = 0.33 for rs2910164). As shown in Table 3, if we set the test level at 0.002 (0.05/11*2) to consider both the multiple comparisons of 11 SNPs and genotypes of each SNP, two SNPs (rs10759932 and rs2737190) were found to be significantly associated with the risk of tuberculosis. For SNP rs2737190, individuals carrying the AG genotype had a significantly increased risk of either clinical tuberculosis (OR: 1.31, 95% CI: 1.11–1.53) or sputum smear-positive tuberculosis (OR: 1.35, 95% CI: 1.13–1.61). For SNP rs10759932, the association was only significant for clinical tuberculosis, where the TC/CC carrier had a 27% increased risk (OR: 1.27, 95% CI: 1.09–1.46).

Stratification analysis revealed that the effects of genetic variations on tuberculosis were more evident among non-smokers. Two SNPs of rs10759932 and rs2737190 remained significant after correcting for multiple comparisons among non-smokers (Table 4).

### Prognosis analysis

We followed the treatment outcomes of all tuberculosis cases. Among the cases, 874(54.6%) were cured, 480(30.0%) completed treatment, 57(3.6%) failed to be treated, and 190(11.9%) defaulted. We categorized the outcomes as successful (cured or completed treatment) and unsuccessful. Single SNP analysis showed that rs3819024 in IL-17A and rs763780 in IL-17F were significantly associated with the treatment outcomes of tuberculosis. For the SNP rs3819024, individuals carrying the AG genotype were likely to have a decreased risk of treatment failure when compared with the AA genotype, with an adjusted RR of 0.56 (95% CI: 0.31–1.00) (Table 5). The dominant model showed a 41% decreased risk among individuals carrying variant genotypes (AG/GG), with the adjusted RR of 0.59 (95% CI: 0.34–0.99, P = 0.045). For the SNP rs763780, individuals carrying the TC genotype were likely to have a significantly increased risk of treatment failure when compared with the TT genotype (adjusted RR: 1.84, 95% CI: 1.05–3.14) (Table 5). The dominant model showed a 77% increased risk among individuals carrying variant genotypes (TC/CC), with an adjusted RR of 1.77(95% CI: 1.02–2.99). However, these differences were not significant after Bonferroni correction.

### Linkage analysis and haplotype construction

**Figure 2:**
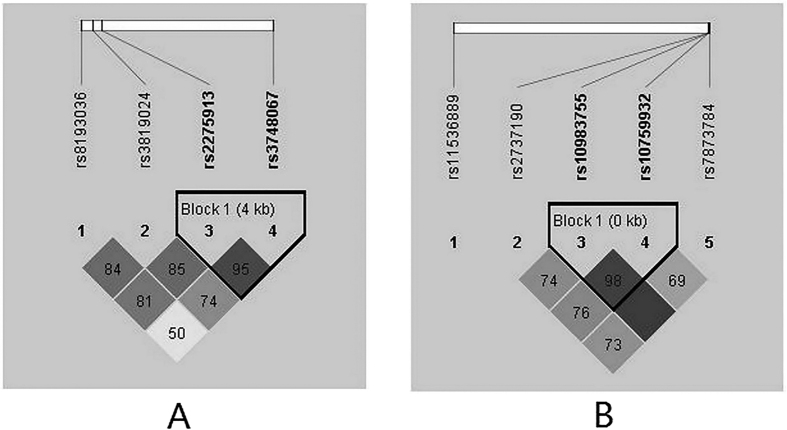
Graphical representation of the SNP locations and LD structure. The SNP distribution and haplotype block structure across IL-17A and TLR4 genes are shown. The measure of LD (D’) among all possible pairs of SNPs is shown graphically according to the shade of color (**A/B**), where white represents very low D’, and dark represents very high D’. The numbers in squares are D’ values (D’ × 100).

To better understand the genetic associations, the linkage disequilibrium (LD) and haplotype blocks were further assessed. LD analysis was carried out on four SNPs of IL-17A and five SNPs of TLR4. Figure 2 displays the LD plot of SNPs on the same chromosome. With a D’ ≥0.95, two SNPs (rs2275913 and rs3748067) of IL-17A on chromosome 6, as well as two SNPs (rs10983755 and rs10759932) of TLR4 on chromosome 9, were in relatively strong linkage disequilibrium with one another. Thus, we performed a haplotype analysis based on these four SNPs. As shown in Table 6, compared with the common haplotype rs10983755G–rs10759932T, rs10983755G–rs10759932C had a significantly increased risk of tuberculosis (OR: 3.43, 95% CI: 2.34–5.05). This increased risk remained significant after Bonferroni correction for multiple comparisons. No significant haplotypes were found be related to the treatment outcome (data not shown).

## Discussion

The magnitude and complexity of the human immune response to mycobacteria have historically been underestimated^23^. It is vital to determine whether those who remain healthy have a genetically endowed high level of resistance to tuberculosis or whether the resistance is affected by environmental or other exogenous factors^24^. The genome-wide association study (GWAS) identified several susceptibility loci for tuberculosis in sub-Saharan African, Russian and Moroccan populations^25^,^26^,^27^. However, the follow-up studies reported conflicting results^28^.

In the present study, we explored the genetic polymorphisms of IL-17, TLR4 and miR-146a in association with pulmonary tuberculosis in a Chinese Han population. To our knowledge, this is the first study revealing the effect of genetic variations of rs10759932 and rs2737190 of TLR4 on the risk of tuberculosis. Haplotype analysis found an increased risk for tuberculosis among individuals carrying TLR4 rs10983755G–rs10759932C. Moreover, we found that SNPs of rs3819024 in IL-17A and rs763780 in IL-17F might be weakly related to the tuberculosis prognosis.

Cytokine secretion is initiated by different immune cells interacting with bacteria^29^. IL-17 acts as a pro-inflammatory cytokine by recruiting granulocytes to the sites of infection^17^. Previous studies have suggested the association between genetic polymorphisms of IL-17A/IL-17F and susceptibility to tuberculosis but with inconsistent results^18^,^30^,^31^,^32^. Du *et al*. observed that the rs763780-CC polymorphisms of the IL-17F gene were more likely to have an increased risk^30^. Ocejo-Vinyals *et al*. investigated the IL-17A rs2275913 polymorphisms and suggested that the GG genotype was related to an increased risk of tuberculosis^18^. Shi *et al*. genotyped rs2275913 and rs3748067 in IL-17A and rs763780 in IL-17F and found that the CC genotype of rs763780 was associated with an increased risk of tuberculosis^32^. Peng *et al*. conducted a study in a Chinese population and found that those carrying the CT/TT genotype of rs763780 were more susceptible to tuberculosis, but no significant association was found for rs2275913^31^. The discrepancies between these results may be due to the different ethnicities, study design and sample sizes^32^.

TLR4 is expressed on the plasma membrane and bind lipoprotein or lipid components of bacteria, and it may sense and simultaneously recognize various MTB-encoded factors. TLR4 signaling may have a critical function in fine tuning inflammation during chronic mycobacterial infection^33^. The SNP rs10759932 is located in the 5′ flanking region of the TLR4 gene^34^. It has been reported to be associated with the risk of precancerous lesions in the stomach^35^, gastric carcinogenesis^34^ or prostate cancer^36^. In contrast to the findings of a study in a Sudanese population^37^, we found that variations of this SNP were related to an increased risk of tuberculosis. The SNP rs2737190 is located in the 5′-UTR of TLR4 gene. As 5′-UTR influences the translation of regulatory proteins, modulation of 5′-UTR activity plays a role in the development or progress of specific forms of disease^38^. Zhou *et al*. have observed that the G allele was more frequent among preterm gram-negative bacterial infection neonates with a 32% increased risk^39^. We first explored the effect of the polymorphism at this locus on susceptibility to pulmonary tuberculosis. Our findings support the hypothesis that genetic polymorphisms of the TLR4 gene affect the host’s susceptibility to infectious diseases.

MiR-146a has been previously described as a negative regulator of the immune response and its systemic down-regulation may be associated with the exacerbated inflammatory response in tuberculosis patients^40^. Pre-miR-146a C/G polymorphism, designated rs2910164, is encoded on chromosome 5q33 and located in the precursor stem region, +60 relative to the first nucleotide of pre-miR-146a, opposite the mature miR-146a sequence^41^. The change from the G:U pair to the C:U mismatch in the stem structure of the miR-146a precursor might reduce the stability of the pri-miR, the efficiency of processing pri-miR into pre-miR, or processing pre-miR into mature miR^42^. Previous studies indicated that miR-146a rs2910164 was related to an altered risk of colorectal cancer^43^, breast cancer or ovarian cancer^44^. To date, two studies have described the association between this SNP and tuberculosis^45^,^46^. One was performed in a Kazak population^45^, and another was conducted in a Tibetan/Han population^46^. However, our study did not replicate the previous significant findings in the Chinese Han population. This difference might be attributed to the variations in allelic frequencies of genetic polymorphisms, and therefore, it is not surprising that the genetic association analyses yielded conflicting results in different populations^47^.

Haplotype-based methods offer a powerful approach to disease gene mapping, based on the association between causal mutations and the ancestral haplotypes from which they arose^48^. In this study, we constructed an LD analysis and identified SNPs of IL-17A and TLR4 in a Chinese Han population. Our data showed a combined effect of rs2275913 together with rs3748067 on the risk of tuberculosis. Additionally, a LD was found between rs10983755 and rs10759932, contributing to the susceptibility of tuberculosis. LD is a concept of statistical correlation between alleles segregated at two or more loci. Population genetic factors can produce LD through a variety of processes such as natural selection, strong genetic drift, admixture and new mutations^49^. The association between each mutant allele and its ancestral haplotype is disrupted only by mutation and recombination in subsequent generations^48^. Further approaches should be carried out to identify the responsible functional SNPs in the LD areas where we identified risk haplotype alleles.

There are several limitations in this study. First, we purposely selected functional SNPs in the IL-17A, IL-17F and TLR4 gene. Although the analysis of the Encyclopedia of DNA Elements (ENCODE) as implemented in Regulome DB indicated that some SNPs might influence the binding of specified transcription factors, their real functions were not proven with experimental evidence. Further work with both knockout and overexpression models is likely to be the most fruitful approach for understanding the mechanisms through which these variants influence the risk of tuberculosis. Second, due to the weak effect of a single genetic polymorphism, other genes in the immunity pathway, together with environmental factors, should also be considered.

## Conclusions

Taken together, our results suggest that genetic polymorphisms of rs10759932 and rs2737190 in TLR4 gene may play a role in susceptibility to tuberculosis in the Chinese population.

## Additional Information

**How to cite this article**: Wang, M. *et al*. Genetic polymorphisms of IL-17A, IL-17F, TLR4 and miR-146a in association with the risk of pulmonary tuberculosis. *Sci. Rep.* **6**, 28586; doi: 10.1038/srep28586 (2016).

## Figures and Tables

**Figure 1:**
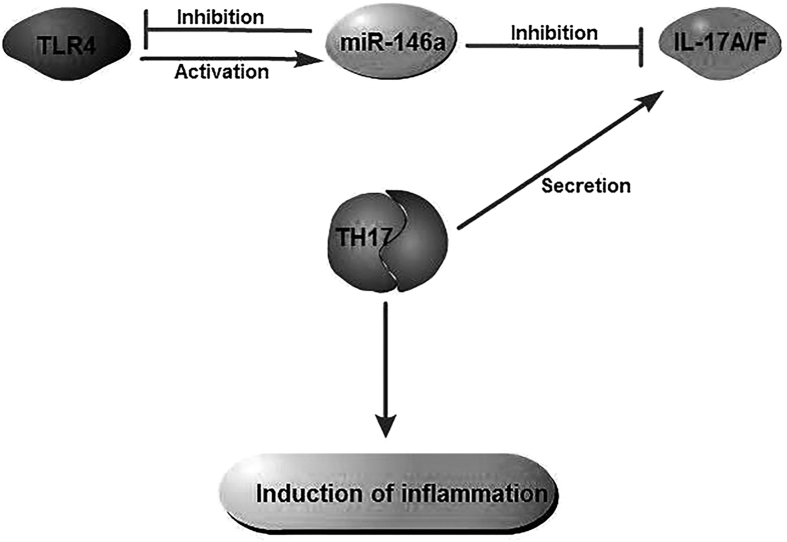


**Table 1:** Primers and probes designed for genotyping.

Gene	SNPs	Primer (5′-3′)	Probe
IL-17A	rs2275913	F-TGAATTTCTGCCCTTCCCATT	A: FAM-CTTCAGAAGAAGAGATT-MGB
	G > A	R-GGTTCAGGGGTGACACCATTT	G: HEX-TTCAGAAGGAGAGATT-MGB
	rs3819024	F-CCGGAATTGTCTCCACAACAC	G: FAM-AATCTGTGAGGGAAAG-MGB
	A > G	R-TGTACCTTGATTTTCCATTTGATCTT	A: HEX-AGGAATCTGTGAGGAAA-MGB
	rs8193036	F-CTCCTTTCTAGTTCTCATCACTCTCTACTC	G: FAM-CTTTTCTCCATCTTCA-MGB
	C > T	R-TGTTTTGAGGAAGGAATTGAAAATG	A: HEX-CTTTTCTCCATCTCCA-MGB
	rs3748067	F-TGAGTTTTTATTTTACTTGGGCTGAA	G: FAM-TTCTCATACTTAAAGTTC-MGB
	G > A	R-CAACCCAGAAAGGAGCTGATG	A: HEX-TTCTCATACTTAAAATTC-MGB
IL-17F	rs763780	F-GAGAAGGTGCTGGTGACTGTTG	G: FAM-CCTGTCATCCACCGTG-MGB
	T > C	R-CTTCTTCAGCTGAGTGGATATGCA	A: HEX-CCTGTCATCCACCATG-MGB
TLR4	rs1927914	F-GAAGTGCTTGGAGGATATTACAGTAGAA	G: FAM-CTAGGACTTAGCATGCATA-MGB
	T > C	R-GAACTGGCATTTGTAAAGCTTTTAGG	A: HEX-ACTTAGCATACATAATATT-MGB
	rs10759932	F-CCCACAAATGGTGTACAGGAGTT	G: FAM-ATCTTCACCAACGCT-MGB
	T > C	R-TGCAAGCTTCTGCTATGATTAAAAG	A: HEX-CATCTTCACCAACACT-MGB
	rs10983755	F-ACCACAAAATGGTCCCTCACA	G: FAM-CTTGGTTTTTGACACGTT-MGB
	G > A	R-TTCTACTGTAATATCCTCCAAGCACTTC	A: HEX-TTGGTTTTTGACACATTG-MGB
	rs2737190	F-GGAGCATGCCTTATGCACACT	T: FAM-ACCCAAGTAGACACTGT-MGB
	A > G	R-GACCTGTGATGATTAGGGCTGAA	C: HEX-ACCCAAGTAGACACCGT-MGB
	rs7873784	F-AGAACACTTAACATGAGAGGTACCC	C: FAM-TTCATTATACGAACTCTGC-MGB
	C > G	R-GATGAATTAGCTCTAAAGATCAGCTGT	G: HEX-TTCATTATAGGAACTCTGC-MGB
	rs11536889	F-GTTGGGCAATGCTCCTTGA	G: FAM-ATTTTGGGAAGAGTGGAT-MGB
	G > C	R-GAACCCCATTAATTCCAGACACA	C: HEX-CACATTTTGGGAACAGT-MGB
miR-146a	rs2910164	F-GAACTGAATTCCATGGGTTGTGT	G: FAM-TCAGACCTGTGAAATT-MGB
	C > G	R-GCCCACGATGACAGAGATATCC	C: HEX-TCAGACCTCTGAAATT-MGB

**Table 2:** General characteristics of the cases and controls.

Variables	Case (n = 1601) n(%)	Control (n = 1526) n(%)	*t/χ*^*2*^	P
Age(years)				
Mean ± SD	52.1 ± 17.7	52.4 ± 17.0	0.564	0.573
Sex			0.321	0.571
Male	1181(73.8)	1112(72.9)		
Female	420(26.2)	414(27.1)		
Smoking			84.730	<0.001
Never	762(47.6)	976(64.0)		
Ever	839(52.4)	550(36.0)		
Drink			9.065	0.003
Never	1246(77.8)	1117(73.2)		
Ever	355(22.2)	409(26.8)		
Sputum smear test			–	–
Positive	1080(67.5)	–		
Negative	521(32.5)	–		

**Table 3:** Genotype distributions of the eleven SNPs among the cases and controls.

Gene	SNPs	Control (n = 1526) n(%)	Total cases(n = 1601)			Smear-positive cases (n = 1080)		
			n(%)	OR(95%Cl)^a^	P	n(%)	OR(95%Cl)^a^	P
IL-17A	rs2275913							
	GG	450(29.6)	477(31.7)	1		309(30.7)	1	
	GA	741(48.7)	729(48.4)	0.89(0.75–1.06)	0.192	494(49.2)	0.94(0.77–1.13)	0.500
	AA	331(21.7)	301(20.0)	0.79(0.64–0.97)	0.028	202(20.1)	0.82(0.65–1.04)	0.098
	Add			0.89(0.80–0.99)	0.027		0.91(0.81–1.02)	0.106
	Dom			0.86(0.73–1.01)	0.066		0.90(0.75–1.08)	0.249
	Rec			0.85(0.71–1.02)	0.074		0.85(0.70–1.05)	0.129
	G	1641(53.9)	1683(55.8)	1		1112(55.3)	1	
	A	1403(46.1)	1331(44.2)	0.93(0.84–1.02)	0.131	898(44.7)	0.95(0.84–1.06)	0.323
	rs3819024							
	AA	422(27.7)	442(28.2)	1		284(26.8)	1	
	AG	745(48.9)	784(50.0)	0.98(0.83–1.16)	0.816	544(51.4)	1.06(0.88–1.29)	0.547
	GG	358(23.5)	341(21.8)	0.85(0.69–1.04)	0.110	230(21.7)	0.90(0.71–1.13)	0.355
	Add			0.92(0.83–1.02)	0.124		0.95(0.85–1.07)	0.401
	Dom			0.94(0.80–1.10)	0.421		1.01(0.84–1.21)	0.942
	Rec			0.86(0.72–1.02)	0.081		0.86(0.71–1.05)	0.137
	A	1589(52.1)	1668(53.2)	1		1112(52.6)	1	
	G	1461(47.9)	1466(46.8)	0.96(0.87–1.06)	0.376	1004(47.4)	0.98(0.88–1.10)	0.748
	rs8193036							
	CC	783(51.4)	789(51.1)	1		515(49.7)	1	
	CT	621(40.7)	618(40.0)	1.00(0.86–1.17)	0.966	429(41.4)	1.07(0.90–1.27)	0.433
	TT	120(7.9)	137(8.9)	1.22(0.93–1.60)	0.156	92(8.9)	1.26(0.93–1.71)	0.128
	Add			1.06(0.95–1.19)	0.312		1.10(0.97–1.25)	0.135
	Dom			1.04(0.90–1.20)	0.623		1.10(0.94–1.30)	0.246
	Rec			1.22(0.93–1.58)	0.146		1.23(0.92–1.64)	0.172
	C	2187(71.8)	2196(71.1)	1		1459(70.4)	1	
	T	861(28.2)	892(28.9)	1.03(0.92–1.15)	0.580	613(29.6)	1.07(0.94–1.21)	0.300
	rs3748067							
	GG	1094(71.8)	1135(71.2)	1		757(70.5)	1	
	GA	385(25.3)	415(26.1)	1.06(0.89–1.25)	0.528	286(26.6)	1.09(0.90–1.31)	0.369
	AA	45(3.0)	43(2.7)	1.02(0.66–1.58)	0.935	31(2.9)	1.13(0.70–1.83)	0.607
	Add			1.04(0.90–1.19)	0.592		1.08(0.93–1.26)	0.325
	Dom			1.05(0.90–1.23)	0.540		1.09(0.92–1.31)	0.327
	Rec			1.00(0.65–1.55)	0.984		1.11(0.69–1.79)	0.670
	G	2573(84.4)	2685(84.3)	1		1800(83.8)	1	
	A	475(15.6)	501(15.7)	1.01(0.88–1.16)	0.878	348(16.2)	1.05(0.90–1.22)	0.549
IL-17F	rs763780							
	TT	1175(77.0)	1225(77.6)	1		840(79.0)	1	
	TC	318(20.9)	323(20.5)	1.00(0.84–1.20)	0.974	207(19.5)	0.95(0.77–1.16)	0.586
	CC	32(2.1)	31(2.0)	0.91(0.54–1.52)	0.713	16(1.5)	0.67(0.36–1.25)	0.207
	Add			0.99(0.85–1.15)	0.866		0.91(0.76–1.08)	0.269
	Dom			0.99(0.84–1.18)	0.947		0.92(0.76–1.12)	0.397
	Rec			0.91(0.54–1.51)	0.710		0.68(0.36–1.26)	0.219
	T	2668(87.5)	2773(87.8)	1		1887(88.8)	1	
	C	382(12.5)	385(12.2)	0.97(0.83–1.13)	0.690	239(11.2)	0.89(0.75–1.05)	0.162
TLR4	rs10759932							
	TT	779(51.4)	722(45.7)	1		505(47.2)	1	
	TC	597(39.4)	697(44.1)	1.27(1.09–1.48)	0.002^b^	458(42.8)	1.18(1.00–1.40)	0.054
	CC	140(9.2)	161(10.2)	1.25(0.97–1.61)	0.090	107(10.0)	1.20(0.91–1.60)	0.199
	Add			1.17(1.05–1.31)	0.005		1.13(1.00–1.28)	0.053
	Dom			1.27(1.09–1.46)	0.001^b^		1.19(1.01–1.39)	0.037
	Rec			1.12(0.87–1.43)	0.378		1.12(0.85–1.47)	0.430
	T	2155(71.1)	2141(67.8)	1		1468(68.6)	1	
	C	877(28.9)	1019(32.2)	1.17(1.05–1.30)	0.005	672(31.4)	1.13(1.00–1.27)	0.055
	rs2737190							
	AA	557(37.0)	518(32.6)	1		343(32.0)	1	
	AG	690(45.8)	840(52.9)	1.31(1.11–1.53)	0.001^b^	580(54.1)	1.35(1.13–1.61)	0.001^b^
	GG	259(17.2)	231(14.5)	0.95(0.76–1.18)	0.628	150(14.0)	0.93(0.73–1.19)	0.566
	Add			1.03(0.93–1.15)	0.559		1.03(0.92–1.16)	0.601
	Dom			1.21(1.04–1.41)	0.015		1.23(1.04–1.46)	0.015
	Rec			0.81(0.66–0.99)	0.038		0.78(0.62–0.98)	0.030
	A	1804(59.9)	1876(59.0)	1		1266(59.0)	1	
	G	1208(40.1)	1302(41.0)	1.04(0.94–1.15)	0.489	880(41.0)	1.04(0.93–1.16)	0.516
	rs10983755							
	GG	793(52.1)	806(50.7)	1		551(51.5)	1	
	GA	600(39.4)	644(40.5)	1.06(0.91–1.23)	0.450	424(39.6)	1.02(0.86–1.21)	0.829
	AA	128(8.4)	139(8.7)	1.06(0.81–1.38)	0.672	95(8.9)	1.07(0.80–1.44)	0.636
	Add			1.04(0.93–1.17)	0.472		1.03(0.91–1.17)	0.650
	Dom			1.06(0.92–1.22)	0.428		1.03(0.88–1.21)	0.731
	Rec			1.03(0.80–1.34)	0.808		1.07(0.80–1.42)	0.665
	G	2186(71.9)	2256(71.0)	1		1526(71.3)	1	
	A	856(28.1)	922(29.0)	1.04(0.94–1.17)	0.446	614(28.7)	1.03(0.91–1.16)	0.664
	rs7873784							
	GG	1271(83.9)	1310(83.1)	1		876(82.2)	1	
	GC	235(15.5)	256(16.2)	1.07(0.88–1.31)	0.476	181(17.0)	1.13(0.91–1.41)	0.265
	CC	9(0.6)	11(0.7)	1.38(0.56–3.41)	0.489	9(0.8)	1.76(0.68–4.54)	0.244
	Add			1.09(0.91–1.31)	0.362		1.16(0.95–1.42)	0.144
	Dom			1.09(0.89–1.32)	0.411		1.15(0.93–1.43)	0.192
	Rec			1.36(0.55–3.37)	0.505		1.72(0.67–4.44)	0.262
	G	2777(91.7)	2876(91.2)	1		1933(90.7)	1	
	C	253(8.3)	278(8.8)	1.06(0.89–1.27)	0.515	199(9.3)	1.13(0.93–1.37)	0.218
	rs11536889							
	GG	891(58.7)	953(60.2)	1		637(59.5)	1	
	GC	545(35.9)	535(33.8)	0.95(0.81–1.11)	0.506	372(34.7)	0.99(0.83–1.18)	0.911
	CC	83(5.5)	94(5.9)	1.04(0.76–1.43)	0.799	62(5.8)	1.03(0.72–1.47)	0.859
	Add			0.98(0.87–1.11)	0.780		1.00(0.88–1.15)	0.970
	Dom			0.96(0.83–1.11)	0.602		1.00(0.85–1.17)	0.962
	Rec			1.06(0.78–1.45)	0.704		1.04(0.73–1.47)	0.840
	G	2327(76.6)	2441(77.1)	1		1646(76.8)	1	
	C	711(23.4)	723(22.9)	0.97(0.86–1.09)	0.606	496(23.2)	0.99(0.87–1.12)	0.836
miR-146a	rs2910164							
	CC	537(35.4)	550(34.7)	1		387(36.1)	1	
	CG	715(47.2)	775(48.9)	1.08(0.92–1.27)	0.360	505(47.2)	1.01(0.84–1.21)	0.927
	GG	264(17.4)	259(16.4)	0.96(0.77–1.19)	0.691	179(16.7)	0.94(0.74–1.19)	0.620
	Add			1.00(0.90–1.10)	0.934		0.98(0.87–1.10)	0.693
	Dom			1.05(0.90–1.22)	0.567		0.99(0.84–1.17)	0.909
	Rec			0.92(0.76–1.11)	0.378		0.94(0.76–1.16)	0.554
	C	1789(59.0)	1875(59.2)	1		1279(59.7)	1	
	G	1243(41.0)	1293(40.8)	0.99(0.90–1.10)	0.884	863(40.3)	0.97(0.87–1.09)	0.610

^a^OR: odds ratio; CI: confidence interval, adjusted for age, sex, smoking and drinking.

^b^Significant after the Bonferroni correction for multiple comparisons. Add: additive model; Dom: dominant model; Rec: recessive model.

**Table 4:** The association between eleven SNPs and the risk of tuberculosis stratified by smoking.

Gene	SNPs	Never(n = 1738)				Ever (n = 1389)			
		Control(%)	Case(%)	OR(95%Cl)^a^	P	Control(%)	Case(%)	OR(95%Cl)^a^	P
IL-17A	rs2275913								
	GG	290(29.7)	257(36.3)	1		160(29.3)	220(27.5)	1	
	GA	487(49.9)	318(44.9)	0.74(0.59–0.92)	0.007	254(46.4)	411(51.4)	1.12(0.87–1.46)	0.380
	AA	198(20.3)	133(18.8)	0.73(0.55–0.97)	0.028	133(24.3)	168(21.0)	0.88(0.65–1.21)	0.439
	rs3819024								
	AA	268(27.5)	234(31.7)	1		154(28.0)	208(25.1)	1	
	AG	492(50.5)	354(47.9)	0.80(0.64–1.01)	0.057	253(46.0)	430(51.9)	1.25(0.96–1.63)	0.100
	GG	215(22.1)	151(20.4)	0.77(0.58–1.01)	0.063	143(26.0)	190(22.9)	0.96(0.71–1.31)	0.796
	rs8193036								
	CC	502(51.5)	347(47.9)	1		281(51.1)	442(54.0)	1	
	CT	401(41.2)	300(41.4)	1.08(0.88–1.33)	0.458	220(40.0)	318(38.8)	0.91(0.72–1.15)	0.437
	TT	71(7.3)	78(10.8)	1.64(1.15–2.33)	0.006	49(8.9)	59(7.2)	0.83(0.54–1.26)	0.372
	rs3748067								
	GG	696(71.4)	529(70.0)	1		398(72.5)	606(72.4)	1	
	GA	256(26.3)	202(26.7)	1.08(0.87–1.35)	0.484	129(23.5)	213(25.4)	1.02(0.79–1.32)	0.882
	AA	23(2.4)	25(3.3)	1.49(0.83–2.67)	0.181	22(4.0)	18(2.2)	0.66(0.34–1.26)	0.206
IL-17F	rs763780								
	TT	747(76.6)	570(76.2)	1		428(77.8)	655(78.8)	1	
	TC	205(21.0)	165(22.1)	1.06(0.84–1.34)	0.625	113(20.5)	158(19.0)	0.94(0.71–1.24)	0.651
	CC	23(2.4)	13(1.7)	0.70(0.35–1.41)	0.318	9(1.6)	18(2.2)	1.29(0.57–2.95)	0.545
TLR4	rs10759932								
	TT	507(52.3)	323(43.4)	1		272(49.8)	399(47.8)	1	
	TC	372(38.4)	348(46.7)	1.47(1.20–1.81)	<0.001^b^	225(41.2)	349(41.8)	1.04(0.82–1.31)	0.751
	CC	91(9.4)	74(9.9)	1.24(0.88–1.75)	0.214	49(9.0)	87(10.4)	1.23(0.83–1.82)	0.299
	rs2737190								
	AA	363(37.8)	238(31.6)	1		194(35.6)	280(33.5)	1	
	AG	437(45.5)	402(53.3)	1.43(1.15–1.78)	0.001^b^	253(46.4)	438(52.5)	1.17(0.91–1.49)	0.215
	GG	161(16.8)	114(15.1)	1.05(0.78–1.41)	0.757	98(18.0)	117(14.0)	0.80(0.58–1.12)	0.200
	rs10983755								
	GG	514(52.8)	369(48.9)	1		279(50.9)	437(52.4)	1	
	GA	375(38.5)	322(42.6)	1.20(0.98–1.47)	0.074	225(41.1)	322(38.6)	0.89(0.71–1.13)	0.338
	AA	84(8.6)	64(8.5)	1.03(0.72–1.47)	0.867	44(8.0)	75(9.0)	1.09(0.72–1.64)	0.685
	rs7873784								
	GG	812(83.9)	606(81.7)	1		459(83.9)	704(84.3)	1	
	GC	151(15.6)	129(17.4)	1.14(0.88–1.48)	0.332	84(15.4)	127(15.2)	0.99(0.73–1.34)	0.939
	CC	5(0.5)	7(0.9)	1.92(0.60–6.15)	0.270	4(0.7)	4(0.5)	0.89(0.22–3.67)	0.873
	rs11536889								
	GG	561(57.8)	456(61.0)	1		330(60.2)	497(59.6)	1	
	GC	355(36.6)	254(34.0)	0.89(0.73–1.10)	0.276	190(34.7)	281(33.7)	1.04(0.82–1.32)	0.720
	CC	55(5.7)	38(5.1)	0.87(0.56–1.35)	0.538	28(5.1)	56(6.7)	1.32(0.81–2.14)	0.267
miR-146a	rs2910164								
	CC	327(33.8)	266(35.3)	1		210(38.3)	284(34.2)	1	
	CG	471(48.7)	364(48.3)	0.93(0.75–1.16)	0.530	244(44.4)	411(49.5)	1.27(0.99–1.62)	0.057
	GG	169(17.5)	123(16.3)	0.87(0.66–1.16)	0.352	95(17.3)	136(16.4)	1.07(0.77–1.48)	0.693

^a^OR: odds ratio; CI: confidence interval, adjusted for age, sex and drinking.

^b^Significant after the Bonferroni correction for multiple comparisons.

**Table 5:** The association analysis of genetic polymorphisms and treatment outcomes.

Gene	SNP	Success (n%)	Failure (n%)	RR(95%Cl)^a^	P
IL-17A	rs2275913				
	GG	397(30.9)	23(43.4)	1	
	GA	635(49.4)	21(39.6)	0.60(0.33–1.08)	0.089
	AA	253(19.7)	9(17.0)	0.62(0.29–1.32)	0.219
	Add			0.75(0.50–1.10)	0.140
	Dom			0.61(0.35–1.04)	0.069
	Rec			0.82(0.40–1.64)	0.576
	rs3819024				
	AA	376(28.1)	23(41.1)	1	
	AG	680(50.8)	22(39.3)	0.56(0.31–1.00)	0.049
	GG	283(21.1)	11(19.6)	0.64(0.31–1.30)	0.219
	Add			0.75(0.52–1.10)	0.143
	Dom			0.59(0.34–0.99)	0.045
	Rec			0.89(0.46–1.68)	0.719
	rs8193036				
	CC	675(51.3)	23(42.6)	1	
	CT	531(40.3)	23(42.6)	1.21(0.69–2.12)	0.503
	TT	111(8.4)	8(14.8)	2.09(0.95–4.36)	0.067
	Add			1.38(0.93–2.05)	0.107
	Dom			1.36(0.80–2.30)	0.251
	Rec			1.91(0.91–3.86)	0.085
	rs3748067				
	GG	971(71.9)	38(66.7)		
	AG	343(25.4)	18(31.6)	1.29(0.74–2.21)	0.373
	AA	37(2.7)	1(1.8)	0.75(0.10–4.75)	0.769
	Add			1.15(0.71–1.85)	0.581
	Dom			1.24(0.72–2.11)	0.440
	Rec			0.69(0.10–4.38)	0.712
IL-17F	rs763780				
	TT	1056(78.6)	37(66.1)	1	
	TC	260(19.3)	18(32.1)	1.84(1.05–3.14)	0.032
	CC	28(2.1)	1(1.8)	1.06(0.14–6.50)	0.955
	Add			1.52(0.95–2.43)	0.082
	Dom			1.77(1.02–2.99)	0.041
	Rec			0.90(0.12–5.50)	0.918
TLR4	rs10759932				
	TT	622(46.2)	23(41.1)	1	
	TC	594(44.1)	27(48.2)	1.16(0.67–1.98)	0.601
	CC	130(9.7)	6(10.7)	1.29(0.53–3.02)	0.573
	Add			1.14(0.77–1.70)	0.515
	Dom			1.18(0.70–1.98)	0.537
	Rec			1.20(0.51–2.68)	0.675
	rs2737190				
	AA	452(33.6)	18(32.1)	1	
	AG	703(52.2)	28(50.0)	0.98(0.55–1.75)	0.958
	GG	191(14.2)	10(17.9)	1.34(0.62–2.78)	0.449
	Add			1.13(0.76–1.67)	0.552
	Dom			1.06(0.61–1.82)	0.840
	Rec			1.35(0.69–2.60)	0.378
	rs10983755				
	GG	682(50.7)	26(45.6)	1	
	GA	547(40.6)	27(47.4)	1.24(0.73–2.08)	0.431
	AA	117(8.7)	4(7.0)	0.96(0.34–2.63)	0.937
	Add			1.09(0.73–1.62)	0.690
	Dom			1.19(0.71–1.97)	0.501
	Rec			0.87(0.31–2.30)	0.778
	rs7873784				
	GG	1120(83.3)	46(82.1)	1	
	GC	215(16.0)	9(16.1)	1.03(0.51–2.06)	0.924
	CC	9(0.7)	1(1.8)	2.61(0.35–12.18)	0.337
	Add			1.16(0.62–2.17)	0.643
	Dom			1.10(0.56–2.13)	0.780
	Rec			2.60(0.35–12.17)	0.339
	rs11536889				
	GG	811(60.3)	31(55.4)	1	
	GC	453(33.7)	23(41.1)	1.31(0.77–2.21)	0.312
	CC	81(6.0)	2(3.6)	0.66(0.16–2.63)	0.568
	Add			1.07(0.70–1.64)	0.753
	Dom			1.22(0.72–2.03)	0.453
	Rec			0.60(0.14–2.32)	0.464
miR-146a	rs2910164				
	CC	468(34.9)	20(35.7)	1	
	GC	660(49.3)	25(44.6)	0.91(0.51–1.60)	0.737
	GG	212(15.8)	11(19.6)	1.18(0.57–2.38)	0.645
	Add			1.06(0.73–1.54)	0.766
	Dom			0.98(0.57–1.66)	0.930
	Rec			1.25(0.65–2.36)	0.495

^a^RR: rate ratio; CI: confidence interval, adjusted for age and sex.

**Table 6:** The haplotype analysis on the risk of tuberculosis.

Haplotype	Control, n(%)	Case, n(%)	OR(95%Cl)^a^	P
rs2275913-rs3748067				
AG	1401(45.90)	1416(44.22)	1	
GG	1176(38.53)	1285(40.13)	1.12(1.00–1..25)	0.046
GA	469(15.37)	493(15.4)	1.09(0.94–1.27)	0.265
AA	6(0.20)	8(0.25)	1.28(0.42–3.87)	0.667
rs10983755-rs10759932				
GT	2159(70.74)	2156(67.33)	1	
AC	846(27.72)	909(28.39)	1.08(0.96–1.21)	0.198
GC	36(1.18)	120(3.75)	3.43(2.34–5.05)	<0.001^b^
AT	11(0.36)	17(0.53)	1.42(0.65–3.09)	0.375

^a^OR: odds ratio; CI: confidence interval, adjusted for age, sex, smoking and drinking.

^b^Significant after the Bonferroni correction for multiple comparisons.
